# Liver MRI proton density fat fraction inference from contrast enhanced CT images using deep learning: A proof-of-concept study

**DOI:** 10.1371/journal.pone.0328867

**Published:** 2025-08-08

**Authors:** Md Nasir, Yixi Xu, Kyle Hasenstab, Alekhya Yechoor, Rahul Dodhia, William B. Weeks, Juan Lavista Ferres, Guilherme Moura Cunha

**Affiliations:** 1 AI for Good Lab, Microsoft, Redmond, Washington, United States of America; 2 Department of Mathematics and Statistics, San Diego State University, San Diego, California, United States of America; 3 Department of Radiology, University of Washington, Seattle, Washington, United States of America; Kaohsiung Medical University, TAIWAN

## Abstract

Metabolic dysfunction-associated steatotic liver disease (MASLD) is the most common cause of chronic liver disease worldwide, affecting over 30% of the global general population. Its progressive nature and association with other chronic diseases makes early diagnosis important. MRI Proton Density Fat Fraction (PDFF) is the most accurate noninvasive method for quantitatively assessing liver fat but is expensive and has limited availability; accurately quantifying liver fat from more accessible and affordable imaging could potentially improve patient care. This proof-of-concept study explores the feasibility of inferring liver MRI-PDFF values from contrast-enhanced computed tomography (CECT) using deep learning. In this retrospective, cross-sectional study, we analyzed data from living liver donor candidates who had concurrent CECT and MRI-PDFF as part of their pre-surgical workup between April 2021 and October 2022. Manual MRI-PDFF analysis was performed following a standard of clinical care protocol and used as ground truth. After liver segmentation and registration, a deep neural network (DNN) with 3D U-Net architecture was trained using CECT images as single channel input and the concurrent MRI-PDFF images as single channel output. We evaluated performance using mean absolute error (MAE) and root mean squared error (RMSE), and mean errors (defined as the mean difference of results of comparator groups), with 95% confidence intervals (CIs). We used Kappa statistics and Bland-Altman plots to assess agreement between DNN-predicted PDFF and ground truth steatosis grades and PDFF values, respectively. The final study cohort was of 94 patients, mean PDFF = 3.8%, range 0.2–22.3%. When comparing ground truth to segmented reference (MRI-PDFF), our model had an MAE of 0.56, an RMSE of 0.77, and a mean error of 0.06 (−1.75,1.86); when comparing medians of the predicted and reference MRI-PDFF images, our model had an MAE, an RMSE, and a mean error of 2.94, 4.27, and 1.28 (−4.58,7.14), respectively. We found substantial agreement between categorical steatosis grades obtained from DNN-predicted and clinical ground truth PDFF (kappa = 0.75). While its ability to infer exact MRI-PDFF values from CECT images was limited, categorical classification of fat fraction at lower grades was robust, outperforming other prior attempted methods.

## Introduction

Metabolic dysfunction-associated steatotic liver disease (MASLD) is a chronic liver condition characterized by excessive accumulation of fat in hepatocytes (i.e., steatosis) which may lead to inflammation, fibrosis, cirrhosis, and hepatocellular carcinoma (liver cancer) [1]. MASLD is the most common cause of chronic liver disease worldwide, affecting over 30% of the global general population and up to 75% of obese individuals [1,2]. NAFLD is associated with metabolic syndrome, insulin resistance, type 2 diabetes, cardiovascular disease, and increased mortality [3,4]. Until late in the course of disease, NAFLD can be a silent condition with no specific symptoms. Clinical suspicion of NAFLD, when present, may lead to diagnostic investigation, with liver biopsy being the gold standard for diagnosis and staging. However, liver biopsy is invasive, costly, and prone to sampling errors and complications [5], suggesting a demand for non-invasive imaging biomarkers that can accurately diagnose and quantify hepatic steatosis, as well as monitor its progression or response to treatment [6].

Magnetic resonance image (MRI) proton density fat fraction (PDFF) has emerged as a safe, accurate, and noninvasive quantitative biomarker of liver fat that is highly correlated with biopsy-obtained histological steatosis grades [7,8], and outperforms other noninvasive imaging methods such as ultrasound and computed tomography (CT) [9]. In brief, on MRI-PDFF images, pixel/voxel values represent the percentile fraction of the MR signal attributable to fat that can be averaged for whole organ fat fraction estimation. However, limitations of MRI-PDFF include susceptibility to artifacts and noise, long acquisition time, high costs, and limited access [10]. To address these limitations, researchers have focused on improving the accuracy of other imaging methods like ultrasound [11] or inferring liver PDFF from less costly and more widely available imaging modalities like Computed Tomography (CT) scans [12], which are widely used for evaluation of abdominal pathology in both acute and elective settings. Assessing liver steatosis using CT could potentially overcome the limitations of MRI-PDFF as a screening tool, particularly when considering the high prevalence of NAFLD in the general population. However, while non-contrast CT attenuation values have a linear correlation with MRI-PDFF, most abdominal CT exams are performed with intravenous contrast administration, which affects the inherent attenuation (i.e., brightness) of the liver, and hence, represents a confounder for accurate liver fat estimation. Further, current methods to diagnose and quantify liver fat from imaging modalities other than MRI-PDFF have lower diagnostic performance at lower liver fat fraction ranges (PDFF<20%) [13].

To address these limitations, we explored the feasibility of inferring liver MRI-PDFF directly from contrast-enhanced computed tomography (CECT) using an automated deep learning-based approach with a focus on lower fat fraction ranges.

## Methods

### Study design and population

We conducted a retrospective, cross-sectional study. 171 consecutive living liver donor candidates with scheduled concurrent CECTs and MRIs between April 2021 and October 2022 scheduled as part of their pre-surgical workup were identified. Of these, we selected 151 subjects who were at least 18 years old an who had CECTs of the abdomen and liver MRIs performed no more than 48 hours apart from each other. Exclusion criteria were if imaging artifacts or omissions resulted in preprocessing failure for automated segmentation, their MRIs did not have MRI-PDFF acquisitions, or their CT were performed without intravenous contrast.

### CECT exams

Patients were scanned in fasting state (6 hours) using multidetector CT scanners (GE Healthcare, Waukesha, WI). Images were acquired monoenergetic at 120kV in the axial plane at different slice thicknesses (0.65–1.5 mm) after intra-venous iodine-based contrast administration and analyzed using axial reconstruction of 2.5 mm. Post contrast imaging phases were arterial, portal venous and hepatic venous phase following institutional weight-based dosing protocol with an average injection flow rate of 3 mL/ second.

### MRI exams

Liver MRIs were performed in either 1.5T or 3T scanners (Philips healthcare, Netherlands) using a surface phased array coil. Multiplanar, multisequence acquisitions were performed, including a confounder corrected multi-echo sequence (mDIXON quant) for PDFF estimation. Multiparametric quantitative maps (PDFF, T2* and R2*) were generated online for analysis.

### Image analysis

We used manual quantitative MRI-PDFF analysis as ground-truth for liver fat estimations and to assess the accuracy of automated PDFF extraction. Manual PDFF analysis was performed following a standard of clinical care protocol, as follows: on post-processed PDFF maps, an abdominal imaging clinical fellow (with 1 year experience) supervised by a board-certified radiologist (with 13 years of experience) drew two regions of interest (ROI) in the right lobe and one in left lobe of the liver to extract mean PDFF values; the three ROI PDFF values were averaged to describe each individual patient’s mean manual PDFF. A clinical PACS software (Visage 7, Visage Imaging) was used for this analysis. ROIs were standardized in size and location across all subjects [14].

### Image preprocessing

The image preprocessing pipeline consisted of three primary steps: segmenting CECT and MRIs to obtain liver masks, isolating liver pixel data on CECT and MRIs using their corresponding masks (herein referred to as *cropped liver)* and registering the cropped liver across CECTs and MRIs.

CECT liver segmentation was performed using an open-source deep learning segmentation model (livermask) [15]. Because no pre-trained model existed to segment the liver on MRI-obtained PDFF images, we used an indirect approach by utilizing post-contrast T1-weighted images from the same MRI exam. First, T1-weighted MRIs were resized to the corresponding PDFF image and the liver was segmented from T1-weighted MRIs using a 3D liver segmentation extension of a 2D U-net CNN segmentation tool [16]. Since PDFF and T1 images were part of the same exam (*i.e.*, there was no difference in liver shape), the liver mask was then propagated onto the PDFF-MRI image by registering the whole T1 image to the whole PDFF and then using the obtained transformation to propagate the mask only. Prior to this step, we performed additional denoising of PDFF to reduce the effect of granular noise visible outside the abdomen using the N4 Bias Field Correction algorithm which is typically used to remove intensity inhomogeneity caused by the bias field [17]. Since this method also alters the intensity values of the image, it is only done as an intermediate step to improve the registration of the mask; original PDFF intensities are retained for the subsequent steps.

After propagating the liver mask onto the PDFF domain, we eroded the outer 1 cm of the mask to prevent anatomical fat from causing PDFF overestimation. Using the refined 3D liver masks, we extracted the liver regions from both CECT and PDFF images. To preserve the spatial context during subsequent registration, we applied a 20% padding (10% on each side) in all three dimensions by adding zero-intensity voxels.

The padded liver regions were then resampled to 128 × 128 × 96 volumes to standardize input sizes. These preprocessed images were used for liver segmentation via the deep learning model. Finally, for extracting liver PDFF from the predicted PDFF-MRI images generated by the U-Net model, we applied the liver mask and computed mean and median PDFF values.

**Fig 1:**
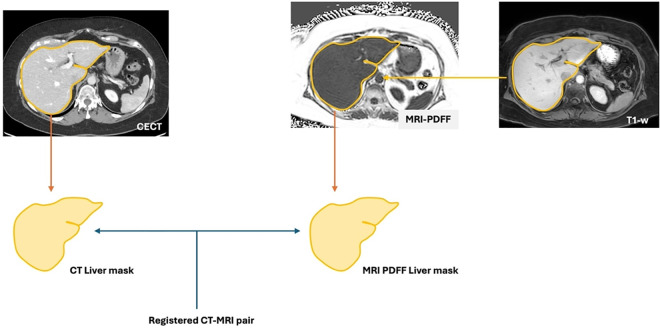
A schematic overview of the preprocessing pipeline.

During the registration process, we performed an additional denoising of the PDFF liver using histogram equalization to enhance image contrast and improve registration quality; however, because PDFF images contain quantifiable information (*i.e.*, each voxel value represents a fat fraction value), once the registration steps were completed, the original PDFF voxels values were used for analysis. Thus, we ended up having paired 3D CECT and PDFF images containing livers only, which represent the source and target of our proposed inference paradigm. All registration steps were performed using ANTs tool and based on affine transformation which allows shearing and scaling in addition to translation and rotation. Fig 1 presents a schematic that illustrates the image preprocessing pipeline.

### U-Net model for inference

CECT images were used as input to a deep neural network (DNN) with 3D U-Net architecture with single channel input and output (128 × 128 × 96 volumes), and the corresponding PDFF images were used as the outputs. This was done after randomly splitting the dataset into two sets: 75% of subjects were used for training and the remaining 25% were used for testing.

For training the neural network model, we explored three different loss functions that measure the distance between the predicted PDFF liver volume pixels and the reference: mean squared error (MSE) loss; a robust version of MSE loss called Huber loss [18] that is less sensitive to large errors; and a weighted combination of MSE and cosine distance loss [12]. We did not observe any noticeable benefits from using the other two losses over the comparatively simpler MSE loss, and, hence used MSE loss for experiments.

Our model was implemented using the MONAI library with PyTorch backend. We used a stochastic gradient descent optimizer for training with a learning rate of 5e-4 and randomly initialized weights. Experimentation was conducted on a machine with 2 NVIDIA Tesla V100 GPUs. Training was performed for 100 epochs with a provision for early stopping if the validation loss did not improve over a period of 10 epochs. In our experiments, the lowest validation loss was achieved after 40 epochs.

### Liver fat fraction predictions and analyses

We computed segmented liver PDFF references from the whole liver PDFF images by computing mean and median PDFF values of the voxels included in the liver mask.

From the predicted PDFF-MRIs generated by the U-Net model, we estimated mean and median PDFF values after applying the liver mask.

In clinical practice, steatosis grades are as follows: Normal (<5%), Mild (5.1–15%), or Moderate/severe (>15.1%) [19]. As a proof of concept to investigate the ability to quantify fat at lower grades overcoming limitations of other methods, categorical analysis was performed by combining normal and mild ranges of steatosis and stratified them into three groups: Lower (<2%), Mid (2–5%), and Upper (5.1–15%).

### Qualitative analysis

DNN-predicted PDFF images were visually compared to the ground-truth PDFF images by [name withheld for blinding purposes]. In addition to visual similarity, we evaluated homogeneity of liver signal intensity, topographic distribution of signal, and presence of imaging artifacts. To represent the 3D volume of these images in 2D, we calculated the average intensity projection (AIP) using the average volume intensities in the axial direction.

### Statistical analysis

To compare mean and median PDFF values from the DNN-predictions, segmented references, and ground truths, we computed two error metrics—mean absolute error (MAE) and root mean squared error (RMSE) — in a pairwise fashion. The error metrics are on the same percentage scale as the fat fractions, and a lower metric value indicates higher similarity between images. MAE aggregates high and low voxel-wise differences in a linear fashion, while RMSE emphasizes larger differences. To compare performance of different comparators (e.g., results of segmented reference vs. ground truth), we calculated mean errors (defined as the error between those approaches) (and 95% confidence intervals (CIs)), for both mean and median approaches. Lower values indicated more consistency across comparators. We used Cohen’s Kappa statistics to assess the agreement between DNN-predicted steatosis categorical grades. Agreement was interpreted as follows: ≤ 0 none, 0.01–0.20 slight, 0.21–0.40 fair, 0.41–0.60 moderate, 0.61–0.80 substantial, and 0.81–1.00 nearly perfect agreement [20]. We used Bland-Altman plots to assess agreement between PDFF values.

### Ethical considerations

On March 1, 2022, the institutional ethics review board of University of Washington approved this study and waived the requirement for informed consent due to the retrospective nature of data collection and analysis (IRB ID STUDY 000015000). Data were collected and handled in accordance with the Health Insurance Portability and Accountability Act. The authors had control of all the data and the information required for development of this paper. All the analyses in this study were performed in accordance with the Declaration of Helsinki. Data was accessed for research between August 1, 2022 – October 31, 2022.

## Results

### Study cohort

**Fig 2:**
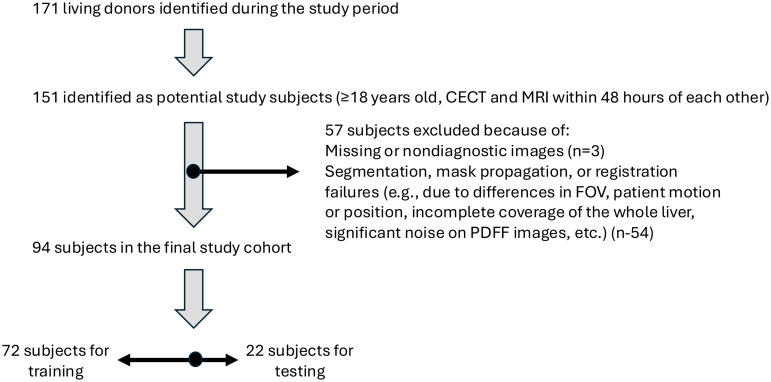
Study population diagram.

Of the 151 selected individuals in the eligible study cohort, 57 were excluded from the dataset due to: missing or nondiagnostic images (n = 3), CECT or MRI images segmentation failure (n = 34), or CECT to MRI liver registration failure (n = 20). Overall, these exclusions were due to technical issues occurring after images acquisition, during image transfer between clinical systems to the research environment or during postprocessing, and hence, with no association to individual patients’ characteristics. Hence, characteristics of excluded patients did not differ from included patients (S1 Table and S2 Table). The final study population consisted of 94 patients. The high number of exclusions may be attributed to use of indirect liver mask propagation in absence of liver segmentation model in PDFF space and high amount of noise artefacts present in input and target both. The demographic information of the individuals is given in Table 1. Fig 2 is a study flow diagram.

**Table 1:** Demographic and ground truth statistics of the study participants.

Variable	Statistics (n = 94)
**Demographic variables**	
Age (Years)	mean: 36.11, SD:10.09, range: 18–56
Sex	counts: (Male: 34, Female: 60)
Weight (kg)	mean: 75.77, SD: 15.11, range: 44–120
**Ground truth**	
Liver fat (%)	mean: 3.87, SD: 3.52, range: 0–22.3

### Qualitative analysis of inferred PDFF images

**Fig 3:**
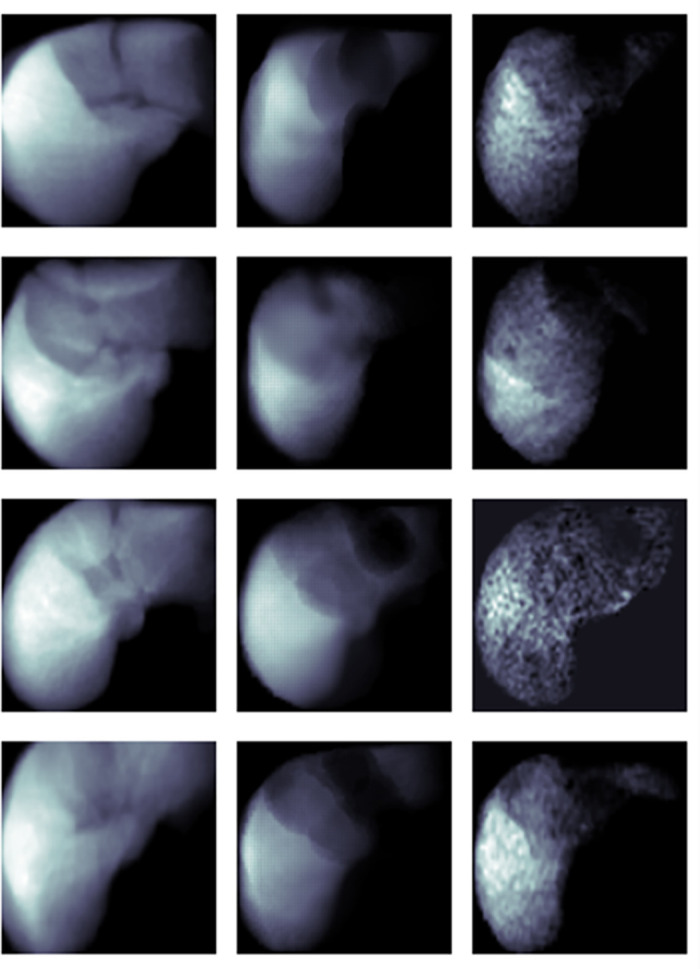
Average intensity projection (AIP) of the 3D volumes: Input CT image (left), PDFF inference as model output (middle), reference PDFF images (right).

Qualitative assessment found that predicted PDFFs had similar signal intensity and signal topography as their references. This particularly held true for large patterns and spots in the liver; however, smaller focal patterns were not as similar. We found some noisy patterns in the predicted PDFF images which were absent both in the reference PDFF and the source CECT images. We interpreted these patterns as artifacts of the CNN-based prediction model attempting to predict less smooth intensity voxels of PDFF from smoother CECT images. Examples of our qualitative analysis of features are shown in Fig 3.

### Performance of fat quantification

The mean averaging method had moderate agreement between ground truths and segmented references with an MAE of 1.53 and an RMSE of 2.62. In contrast, the median averaging method demonstrated a stronger agreement between ground truths and segmented references, with lower MAE and RMSE values of 0.56 and 0.77, respectively. As PDFF values range from 1 to 100, these errors are quite low. Table 2 provides a comparative analysis of mean and median PDFF.

**Table 2:** Comparison between fat fraction (%) from predicted PDFF image and different ground truths using mean and median averaging. The metrics reported are mean absolute error (MAE) and root mean squared error (RMSE).

Comparators	Mean		Median	
	MAE	RMSE	MAE	RMSE
Ground truth – segmented reference fat fraction	1.53	2.62	0.56	0.77
Segmented reference fat fraction- predicted fat fraction	2.91	4.23	2.94	4.27
Ground truth – predicted fat fraction	2.96	4.70	3.17	4.60

Comparing predicted fat fraction to segmented reference using *mean* averaging generated an MAE of 2.91 and an RMSE of 4.23 while *median* averaging yielded an MAE of 2.94 and an RMSE of 4.27. Comparing predicted fat fraction to *manual ground truth* generated an MAE of 2.96 and an RMSE of 4.70 using *mean* averaging and an MAE of 3.17 and an RMSE of 4.60 using median averaging.

**Table 3** shows mean errors and 95% confidence intervals when comparing three fat fraction quantities—predicted fat fraction and the two ground truths. Consistent with the RMSE and MAE metric, we found smaller mean errors and narrower mean error CIs when comparing segmented references and ground truths than when comparing the other comparators.

**Table 3:** Mean errors (calculated as the difference between the two comparators) and 95% confidence interval of mean errors of fat fraction (%) from predicted PDFF image and different ground truths using mean and median averaging.

Comparators	Mean		Median	
	Mean error	CI	Mean error	CI
Ground truth – segmented reference fat fraction	0.20	(−0.96,1.38)	0.06	(−1.75,1.86)
Segmented reference fat fraction- predicted fat fraction	0.64	(−2.12,4.40)	1.28	(−4.58,7.14)
Ground truth – predicted fat fraction	1.84	(−2.83,8.51)	1.34	(−4.40,7.08)

**Fig 4:**
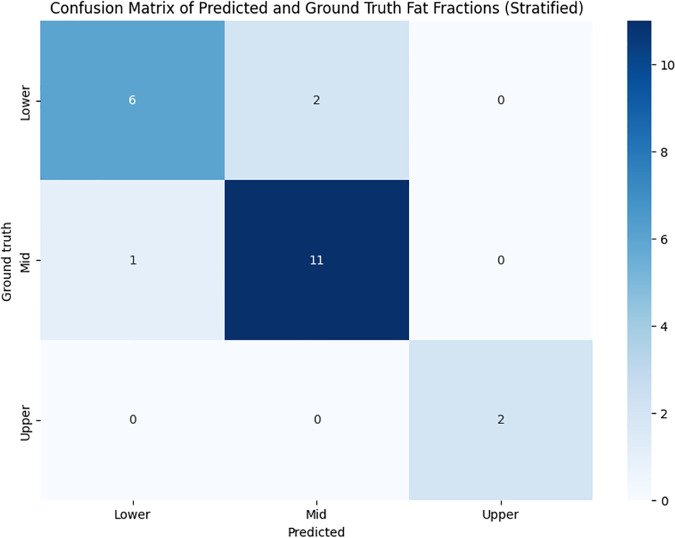
Confusion matrix of the predicted fat fraction using median averaging and the manual ground truth. Each row in the matrix corresponds to a group based on the manual ground truth, labeled as ‘Ground truth’, and each column represents the groups as predicted by the model, labeled as ‘Predicted’. The intersection of a row and a column indicates the number of instances that belong to the respective ‘Ground truth’ and ‘Predicted’ categories.

Fig 4 shows a confusion matrix that compares predicted categories based on median approach to manual ground truth steatosis grades: the rows in the matrix represent manual ground truth (‘Ground truth’) and the columns represent predicted ground truth, with the counts shown in each cell. For example, the top-left cell indicates that there were 6 subjects with ‘Lower’ manual ground truth fat fraction and ‘Lower’ predicted fat fraction. Most subjects were correctly grouped with only 3 errors out of 22 subjects, with an accuracy of 86.4% and Cohen’s kappa = 0.75. Bland-Altman plots show outlier values with higher errors at higher PDFF value (S1 Fig and S2 Fig).

## Discussion

This proof-of-concept work explored the feasibility of using deep learning to infer MRI-PDFF images and calculate fat fractions from CECT images with a focus on lower steatosis grades. We found that inferring MRI-PDFF images from CECT images using deep-learning is possible and, while agreement between absolute PDFF values was not perfect, characterization of liver fat at lower steatosis grades were reliable, differently than other approaches for liver fat quantification using CT.

Despite the different spatial and contrast resolution characteristics of CECT and MRIs, predicted liver PDFF images from CECT images were qualitatively similar to original PDFF images: focal inconsistencies were attributed to DNN artifacts when predicting higher spatial/lower contrast resolution CECT images to lower spatial/higher contrast resolution MRIs. However, for clinical purposes, PDFF generates quantitative values, and, hence, qualitative similarities may be of little clinical relevance. While quantitative error metrics were not low from a technical perspective, given the very large intervals used to grade steatosis in clinical care, they are potentially of little clinical significance. Further, higher error was observed in subjects with higher PDFF values which could potentially be clinically significant. We hypothesize that these errors are due the fact that our study population was skewed towards lower PDFF values, hence, not exposing the model enough to all fat fraction ranges, which may have impacted performance. Based on the results of this proof-of-concept study, however, performance is likely to be improved in further iterations of our approach or in future independent validation studies adopting larger training datasets, ideally with more representative portions of the cohort at higher PDFF values.

When evaluating its ability of categorical stratification at lower fat fraction values our model achieved 86.4% accuracy, with substantial agreement with ground truth measurements. This implies a strength of our method compared to other approaches. A recent metanalysis on the diagnostic accuracy of CT for steatosis shows encouraging results and better performance when diagnosis at least moderate steatosis (PDFF >20%), but limited sensitivity (0.66) when lower steatosis grades are included [13]. For opportunistic screening - and given that most patients with NAFLD in the general population have low to moderate steatosis grades in the low to moderate range [21] - detecting lower ranges may be advantageous as it may relate to early detection and potentially easier reversal to normal than steatosis detected at higher ranges. Using a median averaging as opposed to a mean averaging approach generated more accurate predicted fat fractions and segmented reference fat fractions. This is likely because there is less impact of outliers’ voxel PDFF values (generated from non-liver parenchyma anatomical structures) when using the median averaging approach. While liver segmentation seeks to mitigate the impact of these outlier values by focusing the prediction on the liver, such segmentation is imperfect and can still include outlier values.

In western societies, NAFLD is the fastest growing cause of liver disease and liver cancer [22]. Early intervention can prevent the development of complications, but, as NALFD lacks specific symptoms, diagnosis is not straightforward. Hence, there is demand for accessible and inexpensive ways to screen for NAFLD. While MRI-PDFF is an accurate and reproducible biomarker, MRI use is in clinical practice is limited because due to relatively high cost and limited availability. CT is among the most used imaging modalities for evaluating abdominal pathology, with over 20 million abdominal CT scans being done in the US per year [23], most of which are contrast enhanced. Therefore, CECT offers a possibility of *opportunistic screening* of liver disease. Opportunistic screening in radiology refers to the practice of leveraging imaging data acquired for unrelated clinical indications for diagnosis of incidental conditions [24]. In this context, an accurate, opportunistic diagnosis of MASLD could be made in a patient undergoing CECT for abdominal pain in the emergency department. Prior attempts have been made to infer liver PDFF from CECT; some, like our work, used deep learning-based methods [25–27]. While promising, these techniques relied on using other reference organs or structures to control for the presence of intravenous contrast: for example, a ‘hepatosplenic’ approach [26], in which spleen CECT attenuation was used as a reference point, has been used in model development [10]. While an interesting concept, spleens can be absent or affected by pathology which would impact attenuation values and limit the application of such a model. Our work shows that direct DNN-based PDFF inference from CECT without reliance on reference organs is feasible. Further, prior studies have shown satisfactory performance only when liver fat fraction is in the moderate to severe clinical range, and, therefore, would potentially be less useful in the most common presentation of NAFLD: mild steatosis [21]. Our model accurately stratified patients at lower liver PDFF values, and, therefore, may be useful for screening asymptomatic patients. Since the mere presence of steatosis has clinical relevance [28], detecting patients with mild early NAFLD may have the biggest impact on population health outcomes.

Our work has several limitations. Same day CT and MRI data is not widely available in most centers, and while in a transplant center like ours such data is accessible, the number of living liver transplants performed each year is still relatively small. Further, as a proof of concept study, no commercial tools or turn-key solutions were available at the time of study design, and challenges with imaging data preprocessing resulted in a high number of segmentation-to-registration step failures as we created indirect approaches that added complexity and achieved limited success. The above resulted in a small cohort, which possibly impacted model’s performance. Further, our population was comprised mostly of healthy individuals, without comorbidities, including a history of liver disease. Hence, the generalizability of our results in patients with liver disease cannot be ascertained, though most of such patients would not fall into a population where opportunistic screening might have the highest impact.

## Conclusion

This proof-of-concept study indicates that inferring liver MRI-PDFF directly from contrast-enhanced computed tomography (CECT) using an automated deep learning-based approach with a focus on lower fat fraction ranges is feasible. Categoric predictions were robust, its ability to characterize liver fat content at lower steatosis grades outperforms other methods and makes it potentially clinically useful for screening of the most relevant patient populations. Further model training could improve PDFF estimation accuracy, and machine learning modeling tools advance, they hold the promise of becoming helpful in the early detection and management of NAFLD, ultimately contributing to better patient outcomes and healthcare efficiency.

## Supporting information

S1 Fig Bland-Altman plot of predicted fat fraction and clinical ground truth as reference, using mean averaging. Mean bias = 2.84 and limits of agreement are (−2.83,8.51).
(DOCX)

S2 Fig Bland-Altman plot of predicted fat fraction and clinical ground truth as reference, using median averaging. Mean bias = 1.28 and limits of agreement are (−4.58,7.14).
(DOCX)

S1 Table Demographic and ground truth statistics of included and excluded subjects. (DOCX)

S2 Table Basic demographic, anthropometric and manual liver PDFF analysis of the study population. (DOCX)

## References

[1] Powell EE, Wong VW-S, Rinella M. Non-alcoholic fatty liver disease. Lancet. 2021;397(10290):2212–24. doi: 10.1016/S0140-6736(20)32511-3 33894145

[2] Younossi ZM, Golabi P, Paik JM, Henry A, Van Dongen C, Henry L. The global epidemiology of nonalcoholic fatty liver disease (NAFLD) and nonalcoholic steatohepatitis (NASH): a systematic review. Hepatology. 2023;77(4):1335–47. 36626630 10.1097/HEP.0000000000000004 PMC10026948

[3] Mitrovic B, et al. Non-alcoholic fatty liver disease, metabolic syndrome, and type 2 diabetes mellitus: where do we stand today?. Arch Med Sci. 2022;19:884–94. 37560721 10.5114/aoms/150639 PMC10408022

[4] Yki-Järvinen H. Non-alcoholic fatty liver disease as a cause and a consequence of metabolic syndrome. Lancet Diabetes Endocrinol. 2014;2(11):901–10. doi: 10.1016/S2213-8587(14)70032-4 24731669

[5] Nalbantoglu ILK, Brunt EM. Role of liver biopsy in nonalcoholic fatty liver disease. World J Gastroenterol. 2014;20(27):9026–37. doi: 10.3748/wjg.v20.i27.9026 25083076 PMC4112884

[6] Saadeh S, Younossi ZM, Remer EM, Gramlich T, Ong JP, Hurley M, et al. The utility of radiological imaging in nonalcoholic fatty liver disease. Gastroenterology. 2002;123(3):745–50. doi: 10.1053/gast.2002.35354 12198701

[7] Tang A, Tan J, Sun M, Hamilton G, Bydder M, Wolfson T, et al. Nonalcoholic fatty liver disease: MR imaging of liver proton density fat fraction to assess hepatic steatosis. Radiology. 2013;267(2):422–31. doi: 10.1148/radiol.12120896 23382291 PMC3632805

[8] Wibulpolprasert P, Subpinyo B, Chirnaksorn S, Shantavasinkul PC, Putadechakum S, Phongkitkarun S, et al. Correlation between magnetic resonance imaging proton density fat fraction (MRI-PDFF) and liver biopsy to assess hepatic steatosis in obesity. Sci Rep. 2024;14(1):6895. doi: 10.1038/s41598-024-57324-3 38519637 PMC10960039

[9] Zhang Y, et al. The role of imaging in obesity special feature: review article liver fat imaging — a clinical overview of ultrasound, CT, and MR imaging. Br J Radiol. 2018;91.

[10] Pooler BD, Hernando D, Reeder SB. Clinical Implementation of a Focused MRI Protocol for Hepatic Fat and Iron Quantification. AJR Am J Roentgenol. 2019;213(1):90–5. doi: 10.2214/AJR.18.20947 30917020 PMC6764903

[11] Seneviratne N, Fang C, Sidhu PS. Ultrasound-based hepatic fat quantification: current status and future directions. Clin Radiol. 2023;78(3):187–200. doi: 10.1016/j.crad.2022.10.003 36411088

[12] Wang K, Cunha GM, Hasenstab K, Henderson WC, Middleton MS, Cole SA, et al. Deep Learning for Inference of Hepatic Proton Density Fat Fraction From T1-Weighted In-Phase and Opposed-Phase MRI: Retrospective Analysis of Population-Based Trial Data. AJR Am J Roentgenol. 2023;221(5):620–31. doi: 10.2214/AJR.23.29607 37466189 PMC13173666

[13] Haghshomar M, Antonacci D, Smith AD, Thaker S, Miller FH, Borhani AA. Diagnostic Accuracy of CT for the Detection of Hepatic Steatosis: A Systematic Review and Meta-Analysis. Radiology. 2024;313(2):e241171. doi: 10.1148/radiol.241171 39499183

[14] Campo CA, Hernando D, Schubert T, Bookwalter CA, Pay AJV, Reeder SB. Standardized Approach for ROI-Based Measurements of Proton Density Fat Fraction and R2* in the Liver. AJR Am J Roentgenol. 2017;209(3):592–603. doi: 10.2214/AJR.17.17812 28705058 PMC5639884

[15] Pedersen A, Frutos JP de. Andreped/livermask: v1.5.0. Zenodo. 2023. doi: 10.5281/zenodo.7967326

[16] Wang K, et al. Automated CT and MRI liver segmentation and biometry using a generalized convolutional neural network. Radiol Artif Intell. 2019;1:180022. 32582883 10.1148/ryai.2019180022 PMC7314107

[17] Tustison NJ, Avants BB, Cook PA, Zheng Y, Egan A, Yushkevich PA, et al. N4ITK: improved N3 bias correction. IEEE Trans Med Imaging. 2010;29(6):1310–20. doi: 10.1109/TMI.2010.2046908 20378467 PMC3071855

[18] Huber PJ. Robust Estimation of a Location Parameter. Ann Math Statist. 1964;35(1):73–101. doi: 10.1214/aoms/1177703732

[19] Cunha GM, Thai TT, Hamilton G, Covarrubias Y, Schlein A, Middleton MS, et al. Accuracy of common proton density fat fraction thresholds for magnitude- and complex-based chemical shift-encoded MRI for assessing hepatic steatosis in patients with obesity. Abdom Radiol (NY). 2020;45(3):661–71. doi: 10.1007/s00261-019-02350-3 31781899 PMC8108484

[20] McHugh ML. Interrater reliability: the kappa statistic. Biochem Med (Zagreb). 2012;22(3):276–82. doi: 10.11613/bm.2012.031 23092060 PMC3900052

[21] Makker J, Tariq H, Kumar K, Ravi M, Shaikh DH, Leung V, et al. Prevalence of advanced liver fibrosis and steatosis in type-2 diabetics with normal transaminases: A prospective cohort study. World J Gastroenterol. 2021;27(6):523–33. doi: 10.3748/wjg.v27.i6.523 33642826 PMC7896434

[22] Huang DQ, El-Serag HB, Loomba R. Global epidemiology of NAFLD-related HCC: trends, predictions, risk factors and prevention. Nat Rev Gastroenterol Hepatol. 2021;18:223–38. 33349658 10.1038/s41575-020-00381-6 PMC8016738

[23] Mahesh M, Ansari AJ, Mettler FA. Patient Exposure from Radiologic and Nuclear Medicine Procedures in the United States and Worldwide: 2009–2018. Radiology. 2023;307: e221263. 10.1148/radiol.221263 PMC10050133 36511806

[24] Pickhardt PJ, Summers RM, Garrett JW, Krishnaraj A, Agarwal S, Dreyer KJ, et al. Opportunistic Screening: Radiology Scientific Expert Panel. Radiology. 2023;307(5):e222044. doi: 10.1148/radiol.222044 37219444 PMC10315516

[25] Guo Z, Blake GM, Li K, Liang W, Zhang W, Zhang Y, et al. Liver Fat Content Measurement with Quantitative CT Validated against MRI Proton Density Fat Fraction: A Prospective Study of 400 Healthy Volunteers. Radiology. 2020;294(1):89–97. doi: 10.1148/radiol.2019190467 31687918

[26] Pickhardt PJ, Blake GM, Graffy PM, Sandfort V, Elton DC, Perez AA, et al. Liver Steatosis Categorization on Contrast-Enhanced CT Using a Fully Automated Deep Learning Volumetric Segmentation Tool: Evaluation in 1204 Healthy Adults Using Unenhanced CT as a Reference Standard. AJR Am J Roentgenol. 2021;217(2):359–67. doi: 10.2214/AJR.20.24415 32936018

[27] Pickhardt PJ, Blake GM, Moeller A, Garrett JW, Summers RM. Post-contrast CT liver attenuation alone is superior to the liver-spleen difference for identifying moderate hepatic steatosis. Eur Radiol. 2024;34(11):7041–52. doi: 10.1007/s00330-024-10816-2 38834787

[28] Tamaki N, Ajmera V, Loomba R. Non-invasive methods for imaging hepatic steatosis and their clinical importance in NAFLD. Nat Rev Endocrinol. 2022;18(1):55–66. doi: 10.1038/s41574-021-00584-0 34815553 PMC9012520

