# Supplementary figures and images for: Liver MRI proton density fat fraction inference from contrast enhanced CT images using deep learning: A proof-of-concept study
Source: PLoS One. 2025 Aug 8;20(8):e0328867. doi: 10.1371/journal.pone.0328867 (PMC12333992; doi:10.1371/journal.pone.0328867)

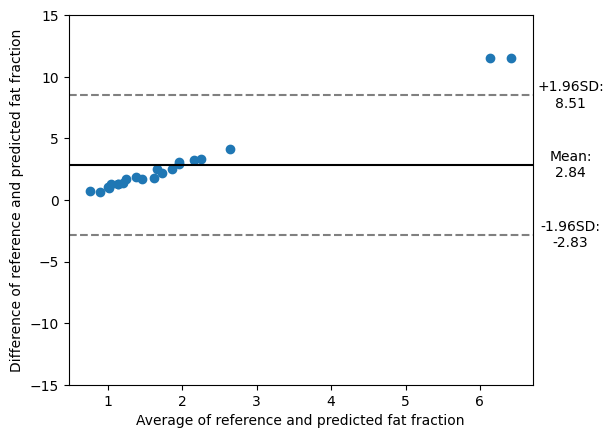

Supplement: S1 Fig — Mean bias = 2.84 and limits of agreement are (−2.83,8.51). (DOCX) [file pone.0328867.s001.docx]

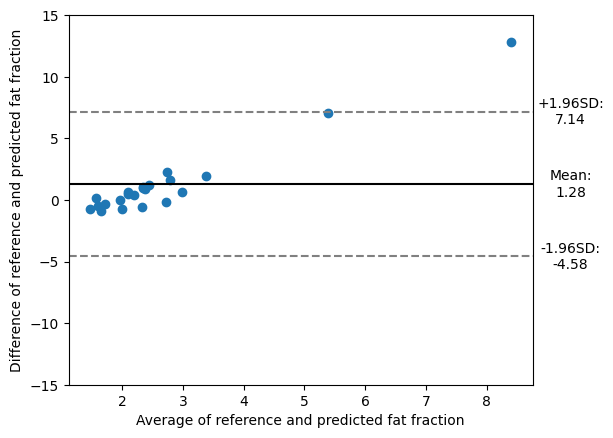

Supplement: S2 Fig — Mean bias = 1.28 and limits of agreement are (−4.58,7.14). (DOCX) [file pone.0328867.s002.docx]
