# Supplementary material for: Liver MRI proton density fat fraction inference from contrast enhanced CT images using deep learning: A proof-of-concept study
Source: PLoS One. 2025 Aug 8;20(8):e0328867. doi: 10.1371/journal.pone.0328867 (PMC12333992; doi:10.1371/journal.pone.0328867)
Supplement: S1 Table — (DOCX) [file pone.0328867.s003.docx]

**Sample**

| Variable | Statistics (n=94) |
| --- | --- |
| Demographic variables | |
| Age (Years) | mean: 34.92, SD:10.53, range: 18-56 |
| Sex | counts: (Male: 34, Female: 60) |
| Weight (kg) | mean: 73.03, SD: 14.88, range: 44-120 |
|  | |
| Ground truth | |
| Liver fat (%) | mean: 3.81, SD: 3.30, range: 0-22.3 |

**Excluded**

| Variable | Statistics (n=57) |
| --- | --- |
| Demographic variables | |
| Age (Years) | mean: 38.14, SD:12.01, range: 18-52 |
| Sex | counts: (Male: 13, Female:32, Not specified:2) |
| Weight (kg) | mean: 80.45, SD: 17.30, range: 52-115 |
|  | |
| Ground truth | |
| Liver fat (%) | mean: 3.97, SD: 3.02, range: 0-18.3 |

**All**

| Variable | Statistics (n=151) |
| --- | --- |
| Demographic variables | |
| Age (Years) | mean: 36.11, SD:10.09, range: 18-56 |
| Sex | counts: (Male: 53, Female: 96, Not specified:2) |
| Weight (kg) | mean: 75.77, SD: 15.11, range: 44-120 |
|  | |
| Ground truth | |
| Liver fat (%) | mean: 3.87, SD: 3.52, range: 0-22.3 |
