# Supplementary material for: Liver MRI proton density fat fraction inference from contrast enhanced CT images using deep learning: A proof-of-concept study
Source: PLoS One. 2025 Aug 8;20(8):e0328867. doi: 10.1371/journal.pone.0328867 (PMC12333992; doi:10.1371/journal.pone.0328867)
Supplement: S2 Table — (DOCX) [file pone.0328867.s004.docx]

| **Study ID** | **Age** | **Sex** | **Height (cm)** | **Weight (Kg)** | **BMI (kg/m²)** | **Liver Right lobe PDFF - ROI 1 (%)** | **Liver Right lobe PDFF - ROI 2 (%)** | **Liver Left lobe PDFF (%)** | **Average liver PDFF (%)** |
| --- | --- | --- | --- | --- | --- | --- | --- | --- | --- |
| QIBM_0002 | 54 | F | 171 | 95 | 32.5 | 2.9 | 3.8 | 3.5 | 3.4 |
| QIBM_0004 | 35 | F | 161 | 70 | 27.0 | 3.6 | 5.2 | 6.2 | 5.0 |
| QIBM_0006 | 34 | M | 177 | 92 | 29.4 | 2.1 | 3.2 | 2.1 | 2.5 |
| QIBM_0008 | 41 | F | 157 | 57 | 23.1 | 1 | 1.3 | 1.3 | 1.2 |
| QIBM_0010 | 41 | F | 162 | 87 | 33.2 | 5.3 | 4.2 | 3.2 | 4.2 |
| QIBM_0012 | 41 | F | 175 | 76 | 24.8 | 0.9 | 0.7 | 0.9 | 0.8 |
| QIBM_0014 | 41 | M | 175 | 101 | 33.0 | 0.8 | 1 | 1.4 | 1.1 |
| QIBM_0016 | 50 | F | 162 | 56 | 21.3 | 1.1 | 1 | 2.5 | 1.5 |
| QIBM_0018 | 45 | M | 177 | 103 | 32.9 | 4 | 4 | 2 | 3.3 |
| QIBM_0020 | 41 | F | 170 | 60 | 20.8 | 3.5 | 3.2 | 2.3 | 3.0 |
| QIBM_0022 | 42 | F | 156 | 73 | 30.0 | 4.1 | 4.9 | 3.9 | 4.3 |
| QIBM_0024 | 40 | F | 172 | 82 | 27.7 | 2.3 | 2 | 2.3 | 2.2 |
| QIBM_0026 | 39 | F | 165 | 56 | 20.6 | 1 | 1 | 2.1 | 1.4 |
| QIBM_0028 | 44 | F | 171 | 70 | 23.9 | 2.9 | 3.2 | 1.8 | 2.6 |
| QIBM_0030 | 43 | M | 172 | 91 | 30.8 | 1.2 | 1.4 | 1.8 | 1.5 |
| QIBM_0032 | 40 | F | 153 | 53 | 22.6 | 6.1 | 7.6 | 5 | 6.2 |
| QIBM_0034 | 58 | F | 165 | 72 | 26.4 | 2.1 | 1.2 | 1.2 | 1.5 |
| QIBM_0036 | 25 | F | 170 | 91 | 31.5 | 1.5 | 1.5 | 1.2 | 1.4 |
| QIBM_0038 | 41 | F | 160 | 81 | 31.6 | 6.2 | 6.8 | 5.5 | 6.2 |
| QIBM_0040 | 44 | F | 170 | 58 | 20.1 | 2.6 | 2.6 | 1.5 | 2.2 |
| QIBM_0042 | 23 | F | 165 | 70 | 25.7 | 4 | 2.9 | 2.1 | 3.0 |
| QIBM_0044 | 34 | M | 177 | 111 | 35.4 | 7 | 5.9 | 5.1 | 6.0 |
| QIBM_0046 | 52 | F | 171 | 67 | 22.9 | 2.7 | 2.7 | 1.5 | 2.3 |
| QIBM_0048 | 45 | F | 172 | 75 | 25.4 | 3.9 | 3.8 | 3.9 | 3.9 |
| QIBM_0050 | 32 | F | 177 | 105 | 33.5 | 4.2 | 5.7 | 4.3 | 4.7 |
| QIBM_0052 | 30 | F | 155 | 63 | 26.2 | 1.5 | 1.5 | 2.2 | 1.7 |
| QIBM_0054 | 38 | F | 167 | 89 | 31.9 | 3.4 | 3.3 | 2.7 | 3.1 |
| QIBM_0056 | 32 | F | 160 | 72 | 28.1 | 9.3 | 7.5 | 8.2 | 8.3 |
| QIBM_0058 | 45 | F | 170 | 75 | 26.0 | 7.5 | 6.9 | 7.3 | 7.2 |
| QIBM_0060 | 52 | M | 169 | 83 | 29.1 | 2 | 2 | 2.4 | 2.1 |
| QIBM_0062 | 23 | F | 167 | 105 | 37.6 | 8.7 | 7.8 | 6.6 | 7.7 |
| QIBM_0064 | 37 | M | 180 | 92 | 28.4 | 0 | 1.2 | 1.5 | 0.9 |
| QIBM_0066 | 41 | F | 168 | 66 | 23.4 | 2.5 | 2.3 | 3.2 | 2.7 |
| QIBM_0068 | 36 | F | 177 | 79 | 25.2 | 4 | 4.4 | 1.8 | 3.4 |
| QIBM_0070 | 40 | M | 172 | 62 | 21.0 | 0.2 | 0.2 | 0.2 | 0.2 |
| QIBM_0072 | 29 | M | 175 | 83 | 27.1 | 7.1 | 5.6 | 5.6 | 6.1 |
| QIBM_0074 | 43 | F | 162 | 65 | 24.8 | 1.2 | 1 | 1.5 | 1.2 |
| QIBM_0076 | 33 | F | 172 | 100 | 33.8 | 2.1 | 4.2 | 3.9 | 3.4 |
| QIBM_0078 | 39 | M | 168 | 62 | 22.0 | 4.2 | 3.8 | 3 | 3.7 |
| QIBM_0080 | 36 | M | 183 | 80 | 23.9 | 3.4 | 3 | 2.3 | 2.9 |
| QIBM_0082 | 34 | M | 180 | 86 | 26.5 | 4.6 | 4.9 | 3 | 4.2 |
| QIBM_0084 | 33 | F | 155 | 62 | 25.8 | 4.1 | 3.4 | 2.9 | 3.5 |
| QIBM_0086 | 44 | F | 168 | 98 | 34.7 | 8.7 | 9.2 | 7.3 | 8.4 |
| QIBM_0088 | 39 | M | 182 | 83 | 25.1 | 4.5 | 5.2 | 3 | 4.2 |
| QIBM_0091 | 37 | F | 174 | 90 | 29.7 | 0.1 | 1.9 | 1.9 | 1.3 |
| QIBM_0093 | 33 | M | 171 | 89 | 30.4 | 7.9 | 6.5 | 6.2 | 6.9 |
| QIBM_0095 | 26 | F | 152 | 59 | 25.5 | 18 | 18 | 12 | 16.0 |
| QIBM_0097 | 40 | M | 175 | 90 | 29.4 | 11 | 13 | 8.4 | 10.8 |
| QIBM_0099 | 35 | M | 169 | 91 | 31.9 | 4.4 | 3.8 | 3.7 | 4.0 |
| QIBM_0101 | 51 | F | 161 | 54 | 20.8 | 1.9 | 1.3 | 2.6 | 1.9 |
| QIBM_0103 | 40 | F | 152 | 73 | 31.6 | 0.2 | 0.5 | 0.2 | 0.3 |
| QIBM_0105 | 24 | M | 179 | 68 | 21.2 | 1.3 | 1.3 | 1.3 | 1.3 |
| QIBM_0107 | 48 | M | 190 | 100 | 27.7 | 6.7 | 20 | 9.9 | 12.2 |
| QIBM_0109 | 36 | M | 188 | 85 | 24.0 | 2.9 | 1.8 | 0.2 | 1.6 |
| QIBM_0111 | 26 | F | 176 | 100 | 32.3 | 1.7 | 2.1 | 1.8 | 1.9 |
| QIBM_0113 | 57 | M | 177 | 84 | 26.8 | 3.7 | 3.9 | 3.5 | 3.7 |
| QIBM_0115 | 24 | M | 175 | 63 | 20.6 | 1.5 | 1.5 | 2 | 1.7 |
| QIBM_0117 | 48 | F | 169 | 80 | 28.0 | 8.6 | 12 | 15 | 11.9 |
| QIBM_0119 | 25 | F | 157 | 49 | 19.9 | 2.1 | 0.9 | 1 | 1.3 |
| QIBM_0121 | 25 | F | 164 | 73 | 27.1 | 3.9 | 4.2 | 3.6 | 3.9 |
| QIBM_0123 | 39 | F | 170 | 79 | 27.3 | 2.8 | 2.7 | 3.1 | 2.9 |
| QIBM_0125 | 33 | F | 162 | 60 | 22.9 | 0 | 0.9 | 1.7 | 0.9 |
| QIBM_0127 | 50 | M | 180 | 85 | 26.2 | 1.7 | 2.2 | 2.1 | 2.0 |
| QIBM_0129 | 45 | F | 173 | 94 | 31.4 | 2.9 | 3.2 | 3 | 3.0 |
| QIBM_0131 | 48 | F | 162 | 59 | 22.5 | 2.3 | 2.9 | 1.4 | 2.2 |
| QIBM_0133 | 35 | F | 165 | 70 | 25.7 | 2.1 | 1.3 | 1.3 | 1.6 |
| QIBM_0135 | 52 | F | 167 | 65 | 23.3 | 1.9 | 1.8 | 3.1 | 2.3 |
| QIBM_0137 | 45 | M | 174 | 97 | 32.0 | 10 | 9.7 | 6.4 | 8.7 |
| QIBM_0139 | 36 | M | 172 | 100 | 33.8 | 6.2 | 6.2 | 6.3 | 6.2 |
| QIBM_0141 | 30 | M | 185 | 83 | 24.3 | 3.7 | 3.3 | 2.5 | 3.2 |
| QIBM_0143 | 36 | F | 174 | 77 | 25.4 | 4.2 | 3.5 | 3.6 | 3.8 |
| QIBM_0145 | 59 | F | 160 | 10 | 3.9 | 1.7 | 1.5 | 3.1 | 2.1 |
| QIBM_0147 | 49 | F | 152 | 53 | 22.9 | 2.9 | 3.2 | 2.6 | 2.9 |
| QIBM_0149 | 36 | F | 165 | 68 | 25.0 | 13 | 14 | 9.7 | 12.2 |
| QIBM_0151 | 57 | F | 157 | 64 | 26.0 | 3.6 | 4 | 3.3 | 3.6 |
| QIBM_0153 | 39 | F | 160 | 52 | 20.3 | 1.1 | 1.2 | 0.2 | 0.8 |
| QIBM_0155 | 41 | F | 174 | 72 | 23.8 | 1.7 | 1.2 | 2.5 | 1.8 |
| QIBM_0157 | 29 | M | 193 | 95 | 25.5 | 18 | 19 | 7.2 | 14.7 |
| QIBM_0159 | 28 | M | 182 | 101 | 30.5 | 9.7 | 9.1 | 3.5 | 7.4 |
| QIBM_0161 | 53 | F | 166 | 75 | 27.2 | 4.8 | 4.2 | 2.1 | 3.7 |
| QIBM_0163 | 44 | M | 178 | 102 | 32.2 | 9.7 | 8.9 | 5.9 | 8.2 |
| QIBM_0165 | 47 | F | 175 | 93 | 30.4 | 4.5 | 3.1 | 3 | 3.5 |
| QIBM_0167 | 24 | M | 180 | 61 | 18.8 | 4.2 | 3.6 | 3.3 | 3.7 |
| QIBM_0169 | 51 | F | 171 | 61 | 20.9 | 3.9 | 3 | 3.1 | 3.3 |
| QIBM_0171 | 21 | F | 162 | 44 | 16.8 | 0.9 | 2.3 | 1.8 | 1.7 |
| QIBM_0173 | 49 | F | 170 | 70 | 24.2 | 1.7 | 0.9 | 1.3 | 1.3 |
| QIBM_0175 | 54 | F | 175 | 93 | 30.4 | 1.8 | 2.3 | 1.8 | 2.0 |
| QIBM_0177 | 44 | M | 178 | 106 | 33.5 | 12 | 15 | 8.6 | 11.9 |
| QIBM_0179 | 21 | M | 174 | 61 | 20.1 | 2.3 | 2.1 | 1 | 1.8 |
| QIBM_0181 | 49 | F | 188 | 96 | 27.2 | 3.3 | 4.7 | 3.1 | 3.7 |
| QIBM_0183 | 59 | F | 157 | 71 | 28.8 | 1.9 | 2.6 | 2.4 | 2.3 |
| QIBM_0185 | 47 | F | 161 | 74 | 28.5 | 0.7 | 0.4 | 1.3 | 0.8 |
| QIBM_0187 | 38 | M | 165 | 82 | 30.1 | 8.1 | 9.7 | 9.5 | 9.1 |
| QIBM_0189 | 30 | F | 161 | 52 | 20.1 | 2.7 | 2.2 | 2.1 | 2.3 |
| QIBM_0191 | 45 | M | 181 | 83 | 25.3 | 2 | 2.8 | 2.6 | 2.5 |
| QIBM_0193 | 44 | F | 160 | 71 | 27.7 | 1.2 | 1.5 | 1.8 | 1.5 |
| QIBM_0195 | 25 | F | 165 | 95 | 34.9 | 7.3 | 7.7 | 5.4 | 6.8 |
| QIBM_0197 | 33 | M | 170 | 80 | 27.7 | 1.8 | 5 | 5 | 3.9 |
| QIBM_0199 | 33 | M | 167 | 68 | 24.4 | 1.8 | 1.6 | 2.9 | 2.1 |
| QIBM_0201 | 51 | M | 158 | 63 | 25.2 | 3.4 | 3.9 | 2.4 | 3.2 |
| QIBM_0203 | 28 | M | 177 | 81 | 25.9 | 0.7 | 1.7 | 1 | 1.1 |
| QIBM_0205 | 59 | F | 171 | 72 | 24.6 | 2.6 | 2.7 | 1.7 | 2.3 |
| QIBM_0207 | 29 | M | 188 | 68 | 19.2 | 6.4 | 5.1 | 3.9 | 5.1 |
| QIBM_0209 | 24 | F | 162 | 67 | 25.5 | 0.1 | 0.3 | 0.8 | 0.4 |
| QIBM_0210 | 31 | F | 155 | 68 | 28.3 | . | . | . | . |
| QIBM_0212 | 47 | F | 170 | 55 | 19.0 | 1.5 | 1.6 | 1.6 | 1.6 |
| QIBM_0214 | 33 | M | 183 | 87 | 26.0 | 2.7 | 1.4 | 1.3 | 1.8 |
| QIBM_0216 | 49 | F | 162 | 53 | 20.2 | 2.9 | 2.3 | 3.2 | 2.8 |
| QIBM_0218 | 53 | M | 190 | 96 | 26.6 | 3.2 | 3.2 | 3.2 | 3.2 |
| QIBM_0220 | 42 | F | 170 | 90 | 31.1 | 20 | 17 | 18 | 18.3 |
| QIBM_0222 | 29 | F | 157 | 55 | 22.3 | 4.1 | 3.5 | 3.2 | 3.6 |
| QIBM_0224 | 51 | M | 188 | 99 | 28.0 | 9.2 | 8.9 | 9.4 | 9.2 |
| QIBM_0226 | 25 | F | 162 | 78 | 29.7 | 9.7 | 9.7 | 5.9 | 8.4 |
| QIBM_0228 | 48 | F | 167 | 94 | 33.7 | 1.6 | 1.1 | 0.4 | 1.0 |
| QIBM_0230 | 40 | F | 152 | 69 | 29.9 | 3.9 | 5 | 3.1 | 4.0 |
| QIBM_0232 | 24 | F | 165 | 59 | 21.7 | 5.5 | 4.8 | 4.3 | 4.9 |
| QIBM_0234 | 27 | F | 170 | 61 | 21.1 | 1.1 | 0.8 | 1.7 | 1.2 |
| QIBM_0236 | 34 | F | 157 | 64 | 26.0 | 2.3 | 1.8 | 2.3 | 2.1 |
| QIBM_0238 | 42 | F | 162 | 87 | 33.2 | 13 | 13 | 8 | 11.3 |
| QIBM_0240 | 24 | M | 175 | 73 | 23.8 | 2.9 | 3.9 | 3.2 | 3.3 |
| QIBM_0242 | 31 | M | 173 | 85 | 28.4 | 1.3 | 1.7 | 0.8 | 1.3 |
| QIBM_0244 | 52 | M | 193 | 119 | 31.9 | 4.8 | 4.1 | 4.4 | 4.4 |
| QIBM_0246 | 50 | F | 150 | 70 | 31.1 | 1.5 | 1.5 | 1 | 1.3 |
| QIBM_0248 | 57 | F | 157 | 90 | 36.5 | 20 | 23 | 24 | 22.3 |
| QIBM_0250 | 33 | F | 170 | 90 | 31.1 | 1.5 | 2.4 | 2.8 | 2.2 |
| QIBM_0252 | 21 | M | 177 | 64 | 20.4 | 2.9 | 2 | 2.6 | 2.5 |
| QIBM_0254 | 42 | F | 165 | 80 | 29.4 | 4.5 | 3.5 | 3.9 | 4.0 |
| QIBM_0256 | 24 | F | 163 | 61 | 23.0 | 2.8 | 0.4 | 2.3 | 1.8 |
| QIBM_0258 | 40 | F | 161 | 62 | 23.9 | 0.6 | 1.6 | 1.3 | 1.2 |
| QIBM_0260 | 43 | F | 170 | 94 | 32.5 | 3.4 | 2.5 | 3.7 | 3.2 |
| QIBM_0262 | 23 | F | 167 | 64 | 22.9 | 3.6 | 3.1 | 3.6 | 3.4 |
| QIBM_0264 | 24 | M | 180 | 105 | 32.4 | 0.7 | 1.8 | 1.5 | 1.3 |
| QIBM_0266 | 40 | M | 177 | 87 | 27.8 | 4.3 | 6.8 | 4.4 | 5.2 |
| QIBM_0268 | 28 | M | 179 | 87 | 27.2 | 1.6 | 2.3 | 1.3 | 1.7 |
| QIBM_0270 | 56 | F | 160 | 61 | 23.8 | 8.7 | 10 | 7.5 | 8.7 |
| QIBM_0272 | 21 | M | 180 | 96 | 29.6 | 3.3 | 2.7 | 2.4 | 2.8 |
| QIBM_0274 | 23 | M | 193 | 94 | 25.2 | 2.3 | 2.6 | 2.2 | 2.4 |
| QIBM_0276 | 29 | M | 193 | 75 | 20.1 | 2.2 | 1.8 | 1.8 | 1.9 |
| QIBM_0278 | 30 | M | 195 | 109 | 28.7 | 3.7 | 2.8 | 2.6 | 3.0 |
| QIBM_0280 | 32 | F | 170 | 95 | 32.9 | 4.1 | 4.5 | 2.6 | 3.7 |
| QIBM_0282 | 49 | F | 173 | 76 | 25.4 | 2.5 | 2.5 | 1.8 | 2.3 |
| QIBM_0284 | 56 | F | 150 | 81 | 36.0 | 3.1 | 3.9 | 2.8 | 3.3 |
| QIBM_0286 | 55 | F | 160 | 86 | 33.6 | 3.2 | 2.3 | 2.3 | 2.6 |
| QIBM_0288 | 33 | F | 169 | 61 | 21.4 | 1.3 | 1.9 | 1.6 | 1.6 |
| QIBM_0290 | 45 | F | 159 | 59 | 23.3 | 2.1 | 1.6 | 1.6 | 1.8 |
| QIBM_0292 | 39 | F | 157 | 49 | 19.9 | 0.7 | 0.5 | 1 | 0.7 |
| QIBM_0294 | 54 | F | 176 | 56 | 18.1 | 2 | 2.1 | 1.8 | 2.0 |
| QIBM_0296 | 43 | F | 170 | 70 | 24.2 | 4.7 | 3.8 | 3.2 | 3.9 |
| QIBM_0298 | 38 | F | 169 | 74 | 25.9 | 0.9 | 0.6 | 0.6 | 0.7 |
| QIBM_0300 | 54 | F | 160 | 71 | 27.7 | 1 | 1 | 1.2 | 1.1 |
| QIBM_0302 | 32 | F | 167 | 65 | 23.3 | 3.2 | 3.7 | 3.1 | 3.3 |
